# Altered Chloride Homeostasis Decreases the Action Potential Threshold and Increases Hyperexcitability in Hippocampal Neurons

**DOI:** 10.1523/ENEURO.0172-17.2017

**Published:** 2018-01-23

**Authors:** Andreas T. Sørensen, Marco Ledri, Miriam Melis, Litsa Nikitidou Ledri, My Andersson, Merab Kokaia

**Affiliations:** Experimental Epilepsy Group, Epilepsy Center, Department of Clinical Sciences, Lund University Hospital, Lund 22184, Sweden

**Keywords:** action potential threshold, chloride, eNpHR3.0, GABA_A_ receptors, halorhodopsin, optogenetics

## Abstract

Chloride ions play an important role in controlling excitability of principal neurons in the central nervous system. When neurotransmitter GABA is released from inhibitory interneurons, activated GABA type A (GABA_A_) receptors on principal neurons become permeable to chloride. Typically, chloride flows through activated GABA_A_ receptors into the neurons causing hyperpolarization or shunting inhibition, and in turn inhibits action potential (AP) generation. However, in situations when intracellular chloride concentration is increased, chloride ions can flow in opposite direction, depolarize neurons, and promote AP generation. It is generally recognized that altered chloride homeostasis per se has no effect on the AP threshold. Here, we demonstrate that chloride overload of mouse principal CA3 pyramidal neurons not only makes these cells more excitable through GABA_A_ receptor activation but also lowers the AP threshold, further aggravating excitability. This phenomenon has not been described in principal neurons and adds to our understanding of mechanisms regulating neuronal and network excitability, particularly in developing brain and during pathological situations with altered chloride homeostasis. This finding further broadens the spectrum of neuronal plasticity regulated by ionic compositions across the cellular membrane.

## Significance Statement

Here, we report that chloride loading, either directly via the recording electrode or indirectly by long-lasting optogenetic activation of halorhodopsin, in the hippocampal CA3 region causes a substantial reduction in the action potential (AP) threshold and aberrant GABA type A (GABA_A_) receptor mediated excitatory activity in individual neurons and networks. Since intracellular chloride accumulation occurs in the developing brain and during pathologic conditions, resetting the AP threshold by chloride might represent a novel mechanism for regulating excitability.

## Introduction

Under normal physiologic conditions in the mature brain, activation of GABA type A (GABA_A_) receptors typically generates chloride flow into the neurons toward the chloride equilibrium potential of around −70 mV (Misgeld et al., 1986; Ganguly et al., 2001). However, at higher intracellular concentrations, as seen in the developing (Ben-Ari et al., 1989; Ben-Ari et al., 1997; Gao and van den Pol, 2001) and epileptic (Ben-Ari, 2006; Raimondo et al., 2015) brain, chloride flows in the opposite direction and depolarizes neurons. This increases the probability of generating seizure activity in adult neuronal networks. Recent work has demonstrated that selective light-induced activation of the inward chloride ion pump, halorhodopsin, eNpHR3.0, derived from the halophilic bacterium *Natronomonas pharaonis* (Zhang et al., 2007; Gradinaru et al., 2008; Gradinaru et al., 2010) and designed to hyperpolarize and silence neurons, can also promote aberrant network hyperactivity (Alfonsa et al., 2015). In line with this observation, a significant proportion of hippocampal (Schoenenberger et al., 2016) or cortical neurons (Chuong et al., 2014) expressing halorhodopsin increase their action potential (AP) firing rates during light illumination. Since eNpHR3.0 activation increases the intracellular chloride concentration, the aberrant excitability and increased AP generation was attributed to depolarizing effect of ligand-gated GABA_A_ receptors. However, intracellular chloride may also activate other membrane channels and pumps (Jentsch et al., 2002). We therefore hypothesized that chloride-loading, apart from affecting chloride flow though GABA_A_ receptors, could trigger another mechanism that would increase firing, e.g., by resetting the AP threshold. Here, we demonstrate that chloride overload not only increase the excitability of the neurons by a GABA_A_ receptor-mediated mechanisms (Raimondo et al., 2012; Mahn et al., 2016) but also substantially decreases the AP threshold. We believe that both factors can contribute to hyperexcitable brain conditions.

## Materials and Methods

### eNpHR3.0 expression in hippocampus

Stereotactic injections with 1-μl AAV5-hSyn1-eNpHR3.0-YFP vector (5.14 × 10^12^ vg/ml) were made in female FVB mice (Charles River, four to five weeks of age) housed before and after surgery in standard cages with 12/12 h light/dark cycle s and provided with food and water *ad libitum*. Animals were anesthetized with 1.5–2.5% isoflurane, 0.5 ml of bupivacaine was used as local anesthetic and chlorhexidine was used for wound cleaning. After placement in a stereotactic frame, a small skin incision and bore hole was made above the target region. A pulled glass capillary fitted to a 5-μl Hamilton syringe was lowered to the target region (anteroposterior (AP), −3.2 mm; mediolateral (ML), 3.1 mm from bregma; dorsoventral (DV), −3.6 and −3.2 from dura; 0.5 μl each depth, 0.1 μl/min). The wound was cleaned and closed with tissue glue. All procedures were performed in accordance with standard guidelines on experimental animal welfare and approved by the Malmö/Lund Animal Research Ethics Board (protocol number; MK 47-2015).

### Slice electrophysiology

Field recordings were performed within CA3 stratum pyramidale, whereas whole-cell recordings were performed from CA3 pyramidal cells. In some cases, field and whole-cell recordings were performed simultaneously in CA3 be placing the two pipettes in close proximity. IR-DIC microscopy was used for visual guidance. Data were sampled at 10-20 kHz with EPC-10 amplifier and PATCHMASTER software (HEKA Elektronik). For current-clamp experiments, the clamp was set at 0 pA for both field and whole-cell recordings, unless otherwise stated. For whole-cell voltage-clamp experiments, the clamp was set at −70 mV. Current ramps of 500 or 1000 pA were used to determine voltage-spike threshold, defined as the first voltage point with dV/dt exceeding 10 mV/ms. When determining the voltage-spike threshold of AP arising during optogenetic application, only the most hyperpolarized threshold was included for statistical analysis. Intrinsic membrane properties, including resting membrane potential (RMP), input resistance, and AP threshold, amplitude, peak, and duration (i.e., half amplitude duration) were measured before optogenetic light application. Uncompensated series resistance (range, 8–30 MΩ) was monitored at the start of the recordings. Cells were loaded by chloride ions using whole-cell pipette solutions with high-chloride concentration or K-gluconate for optogenetic approach. Pulled glass pipettes for field recordings contained artificial CSF (ACSF; 1–3 MΩ tip resistance), whereas pipettes for whole-cell recordings contained 122.5 mM K-gluconate, 12.5 mM KCl, 10 mM KOH-HEPES, 0.2 mM KOH-EGTA, 2 mM MgATP, 0.3 mM Na_3_GTP, and 8 mM NaCl (pH 7.2–7.4, 300–310 mOsm, 3–5 MΩ tip resistance). For high-chloride concentration whole-cell patch-clamp experiments, K-gluconate was substituted with KCL (122 mM final concentration; pH 7.3, 310 mOsm, 3–5 MΩ tip resistance). For K-gluconate and high-chloride-containing pipettes, the theoretical predicted chloride-reversal potential (in ACSF and with no light illumination) was estimated to –48.2 and 3.2 mV, respectively, in the whole-cell configuration.

For the optogenetic chloride loading approach, an AAV vector encoding eNpHR3.0 under the human synapsin 1 promoter was injected into the hippocampus. To allow full transgene expression to occur, we waited at least three weeks before processing the tissue for slicing. The animal was briefly sedated with isoflurane and then decapitated. The brain was removed and immersed in ice-cold solution (modified ACSF; MACSF) containing: 75 mM sucrose, 67 mM NaCl, 26 mM NaHCO_3_, 25 mM glucose, 2.5 mM KCl, 1.25 mM NaH_2_PO_4_, 0.5 mM CaCl_2_, and 7 mM MgCl_2_ (pH 7.35, osmolarity 305–310 mOsm, continuously bobbled with 95% O_2_ and 5% CO_2_). Horizontal slices of 300-μm thickness containing the entire hippocampus including entorhinal cortex was cut on a VT1200 Leica Vibratome. Slices were first incubated in MACSF for 30 min at 34°C, and then transferred to an incubation chamber filled with ACSF containing: 119 mM NaCl, 26.2 mM NaHCO_3_, 11 mM glucose, 2.5 mM KCl, 1.0 mM NaH_2_PO_4_, 2.5 mM CaCl_2_, and 1.3 mM MgSO_4_ (pH 7.4, osmolarity 305–310 mOsm, 95% O_2_ and 5% CO_2_, room temperature). After at least 1 h of recovery, slices were transferred to a submerged recording chamber perfused (2–2.5 ml/min) with carbonated (95% O_2_ and 5% CO_2_) ACSF at 32–34°C. Tissue from age-matched naïve animals was processed similarly.eNpHR3.0 was activated by yellow light generated by a mercury light bulb filtered by a 593 ± 40 nM excitation filter and was applied through the water-immerged 40× microscope lenses (7.4 mW/mm^2^). Photo-currents and potentials were measured in a subset of cells by brief application of light, before proceeding with 5-min continuous illumination. Although the appearance of the cells appeared unaltered by light application, we cannot exclude the possibility that cell swelling occurred, since we did not address this experimentally. Depending on the experiments, fast synaptic transmission and/or spike activity was blocked by applying different pharmacological drugs to the ACSF solution; NBQX (5 μM, AMPA receptor antagonist), APV (50 μM, NMDA receptor antagonist), PTX (100 μM, GABA_A_ receptor antagonist), TTX (1 μM, voltage-gated sodium channel antagonist), and VU0463271 (10 μM, KCC2 antagonist). All drugs were purchased from Sigma-Aldrich. For stimulation of perisomatic inhibitory afferents, a stimulation electrode filled with ACSF was placed in CA3 stratum pyramidale in close proximity of the recorded cell. Paired-pulse stimulations with interstimulus interval of 100 ms was applied at 0.067Hz. The stimulation duration and strength were kept at a minimal at around 0.1 ms and 2 μA, respectively. One field/whole-cell recording was obtained per slice, and the slice was subsequently stored in 4% PFA overnight, rinsed in PBS, and mounted on coverslips. A subset of slices was stained for intracellular visualization of biocytin, which was included in the pipette solutions.

### Experimental design and statistical analysis

All data were analyzed off-line using FITMASTER (HEKA Elektronik), Igor Pro (WaveMetrics), and MiniAnalysis (SynaptoSoft) software. Field recordings were re-filtered with a 1000-Hz low pass band filter, and field EPSPs (fEPSPs) exceeding 0.2 mV from baseline were semiautomatically detected according to individually suited parameter settings (due to different noise levels) for each recording, and visually validated. For whole-cell recordings, postsynaptic potentials (PSPs) exceeding 2 mV were automatically detected. Amplitudes and frequencies were expressed in 10- and 60-s bins. PSP strength was calculated as the area under individual PSPs (mV × ms) and expressed in 10- and 60-s bins. The 60-s bins were used for statistical comparison. Data were compiled in Prism7 (GraphPad Software) for statistical testing and data presentation. Two-sided Student’s *t* test, paired *t* test, and one-way ANOVA followed by Dunnett’s *post hoc* test were used wherever appropriate as specifically noted in the Results or Legends for each comparison. The level of significance was set at *p* < 0.05, and all data were expressed as mean ± SEM, unless otherwise stated.

## Results

### Chloride loading resets the AP threshold in CA3 pyramidal cells

**Figure 1.**
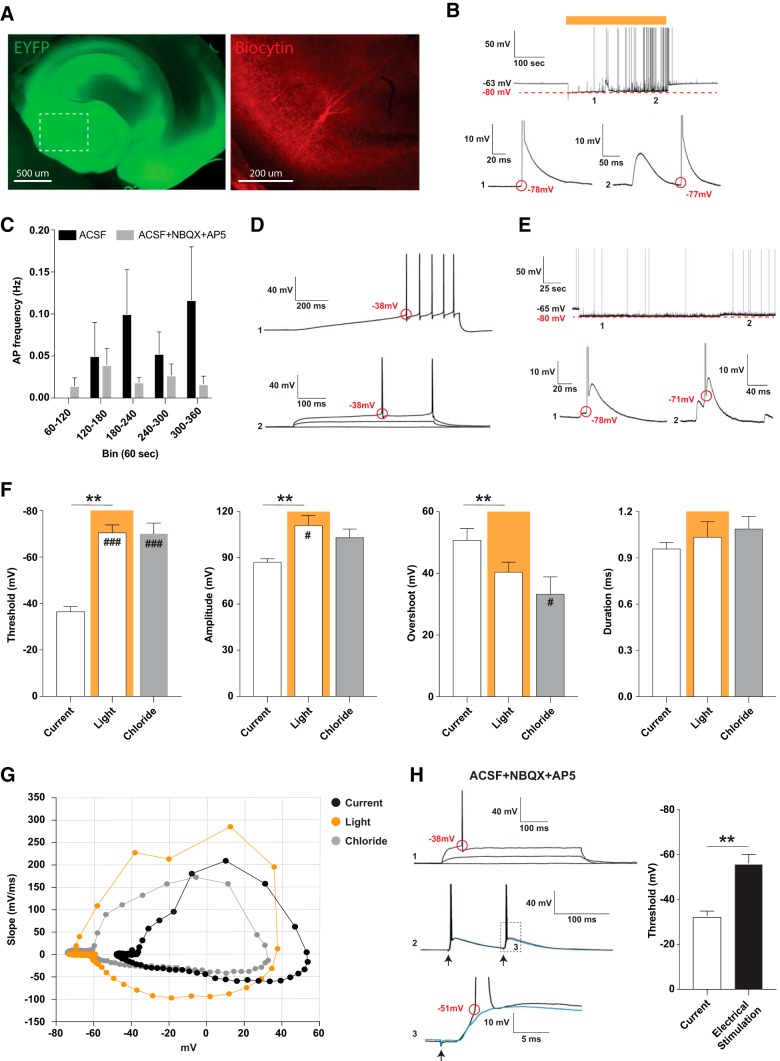
The AP threshold is substantially lowered in CA3 pyramidal cells during conditions of chloride loading. ***A***, Representative slice used for experiments as seen three weeks following viral delivery of AAV-hSyn-NpHR3.0-EYFP into the hippocampus. A biocytin-filled CA3 pyramidal cell is shown within the same slice. ***B***, In ACSF conditions and during 5-min light application, spontaneously APs arises at a much lowered AP threshold. Periods (1–2) from the same trace are magnified and shown below. ***C***, AP frequency during 5-min light for ACSF (*n* = 6 slices from three animals) and ACSF + NBQX + AP5 (*n* = 8 slices from three animals) conditions shown in 60-s bins. ***D***, The same cell as depicted in ***B*** before light application, displaying APs evoked by current injection. Top trace (1) shows a 500-pA ramp depolarization, whereas bottom trace (2) shows a step current depolarization from the same cell. ***E***, Examples of APs elicited spontaneously when a cell was recorded with a pipette containing high concentration of chloride. Periods (1-2) from the same trace are magnified and shown below. ***F***, Basic properties of APs evoked by current ramp injection via patch pipette (current), elicited spontaneously during light application (light), and elicited spontaneously during high-chloride loading via patch pipette (chloride). All three groups were compared by one-way ANOVA with Dunnett’s *post hoc* test and significance is denoted by #, with current serving as the control group, #*p* < 0.05, ###*p* < 0.001; threshold *F*_(2,17)_ = 23.62, *p* < 0.0001; amplitude *F*_(2,17)_ = 4.77, *p* = 0.02; overshoot *F*_(2,17)_ = 3.50, *p* = 0.053; duration *F*_(2,17)_ = 0.66, *p* = 0.53. A paired *t* test (denoted by *) directly compares APs induced by current and light within the same cells; *p < 0.05, **p < 0.01. ***G***, Phase plot showing the slope trajectory during the entire AP cycle of the average APs elicited by current, light, and chloride conditions. Current and light group, *n* = 6 neurons from four animals; chloride group, *n* = 8 neurons from four animals. ***H***, Synaptically evoked AP in the presence of NBQX and AP5. Trace 1, AP threshold determined by step current injection. Trace 2, AP threshold determined by stimulating putative perisomatic GABAergic neurons. Trace 3, Magnified from trace 2, showing two repetitive stimulations with a ∼2.5-s delay from the stimulation artifact to the onset of depolarization. One stimulation give rise to AP (black trace) whereas the other give rise to an EPSP (blue trace). The AP threshold determined for current injection and electrical stimulation is summarized on the right graph, **p < 0.01. In ***B***, ***D***, ***E***, ***H***, red colored text/circle denotes critical values/points of the membrane potential. In magnified traces in ***B***, ***E***, ***H***, the top part of the AP is truncated. In ***C***, ***F***, ***H***, data are shown as mean ± SEM.

To test the hypothesis that increased intracellular chloride concentration alters the AP threshold, we used eNpHR3.0-based optogenetics to load CA3 pyramidal cells with chloride. The viral vector, rAAV-eNpHR3.0, was injected into the mouse hippocampus *in vivo*, and acute hippocampal slices were prepared at least three weeks after. Slices were screened and selected for robust eNpHR3.0 expression throughout the hippocampal network, including dentate gyrus, CA1, and CA3 as well as adjacent entorhinal cortex (Fig. 1*A*). Visual determination of transgene expression (eYFP expression) was confirmed by whole-cell patch-clamp recordings of individual CA3 pyramidal cells. In current-clamp mode (at 0 pA holding) in physiologic ACSF, short pulses of light gave rise to hyperpolarizing photo-potentials (max: −30.2 ± 9.1 mV; steady state: −15.7 ± 5.4 mV; *n* = 8 slices from five animals) and outward photo-currents (max: 616.6 ± 213.6 pA; steady state: 178.2 ± 69.9 pA; *n* = 8 slices from five animals), in agreement with previous observations (Gradinaru et al., 2010; Berglind et al., 2014). To gradually increase chloride loading in CA3 pyramidal neurons over time, we applied constant light to eNpHR3.0 expressing slices for 5 min in the whole-cell configuration. During the course of light application, we observed appearance of spontaneous APs both in normal ACSF (Fig. 1*B*) and ACSF + NBQX + AP5 (Fig. 3*B*) recording conditions. The AP frequency varied, but tended to be higher in ACSF conditions (Fig. 1*C*). Interestingly, these APs seemed to be elicited at a much more hyperpolarized level than normally expected. We therefore measured various parameters for APs provoked by ramp and step-current injections (via patch pipette; Fig. 1*D*,*F*) immediately after membrane break-in for whole-cell configuration. These AP parameters were directly compared with those induced by light application (Fig. 1*B*,*F*). The threshold determined by ramp- or step-depolarization was quite similar within the same cell (Fig. 1*D*), but during light application, the average AP threshold was reduced by 34.0 ± 5.5 mV (*t*_(5)_ = 6.21, *p* = 0.0016, paired *t* test), the absolute AP amplitude was increased by 23.9 ± 5.3 mV (*t*_(5)_ = 4.49, *p* = 0.0065, paired *t* test), the AP overshoot was reduced by 10.3 ± 2.3 mV (*t*_(5)_ = 4.47, *p* = 0.0066, paired *t* test), while the AP width remained unchanged: 0.96 ± 0.04 (current) versus 1.03 ± 0.10 (light) ms (*t*_(5)_ = 0.61, *p* = 0.57, paired *t* test). These findings were unexpected since the AP threshold is almost entirely controlled by voltage-gated sodium channels, and their activation range is normally considered quite narrow around −40 mV (Naundorf et al., 2006; Kole and Stuart, 2008), including in CA3 pyramidal cells (Hemond et al., 2008).

### Chloride loading by whole-cell recording pipette

To exclude that the chloride-induced AP threshold changes were not due to the optogenetic approach per se, we performed a set of experiments on CA3 pyramidal cells with recording pipettes filled with a solution containing high concentration of chloride (122 mM). In the presence of ACSF + NBQX + AP5, we then step-hyperpolarized the membrane potential from −66.1 ± 2.9 to −80.9 ± 2.6 mV in current clamp mode to imitate eNpHR3.0-induced hyperpolarization of the neurons. In such settings, we observed spontaneous APs arising at a threshold of −70.0 ± 5.0 mV (Fig. 1*E*,*F*), quite similar to that observed in eNpHR3.0 loading experiments. In agreement, a phase plot confirmed the decreased threshold and showed generally faster slope velocities throughout the AP cycle during light- and pipette-loading experiments (Fig. 1*G*).

To further rule out whether the membrane excitability of CA3 pyramidal neurons was altered by AAV infection and/or transgene eNpHR3.0 expression per se (Jackman et al., 2014), we compared some basic membrane properties of eNpHR3.0 infected cells with those of naïve cells. None of the compared parameters differed [RMP: −76.1 ± 1.5 vs −73.7 ± 1.8 mV, *t*_(11)_ = 0.99, *p* = 0.34; Ri: 196.9 ± 24.5 vs 206.1 ± 36.7 MΩ, *t*_(11)_ = 0.22, *p* = 0.83; AP threshold (rheobase): 344.6 ± 65.2 vs 468.5 ± 129.5 pA, *t*_(9)_ = 0.90, *p* = 0.39; AP threshold: −35.4 ± 0.9 vs −32.7 ± 1.5 mV, *t*_(9)_ = 1.59, *p* = 0.15; AP amplitude: 87.8 ± 1.5 vs 78.6 ± 6.6 mV, *t*_(9)_ = 1.47, *p* = 0.18; AP spike width: 1.01 ± 0.05 vs 0.99 ± 0.08 ms, *t*_(9)_ = 0.21, *p* = 0.84; eNpHR3.0: *n* = 6-8 slices from five animals versus naïve: *n* = 5 slices from two animals; Student’s *t* test].

We also sought to exclude the possibility that a light-induced hyperpolarization of the membrane potential by itself could change the relative fraction of closed voltage-gated sodium channels toward the open state and thereby lower the AP threshold. We therefore, using our standard K-gluconate pipette solution, hyperpolarized the membrane potential close to −90 mV, and then instantly applied depolarizing steps to determine the AP threshold. During these conditions, the AP threshold remained unchanged (-34.0 ± 0.7 mV @ −90 mV vs −32.2 ± 0.9 mV @ RMP, *t*_(26)_ = 1.57, *p* = 0.13, Student’s *t* test). Thus, it is unlikely that resetting of the AP threshold was caused by transgene expression or the hyperpolarized membrane potential per se, but rather was due to chloride loading. These data together suggested that intracellular chloride accumulation could directly or indirectly decrease the AP threshold.

Next question we asked was how these spontaneous APs were elicited. As expected, spontaneous APs were not observed during light in ACSF + TTX conditions (*n* = 10 slices from 4 animals), confirming that they were generated by voltage-sensitive sodium channels. But were they induced by synaptic inputs? Since they were absent in ACSF + NBQX + AP5 + PTX (*n* = 7 slices from three animals), but present in ACSF alone and ACSF + NBQX + AP5 conditions, it appeared that synaptic activation of GABA_A_ receptors was sufficient to trigger these APs. We focused on GABA release from CA3 perisomatic GABAergic interneurons, shown to generate subthreshold EPSP and even APs in principal cells (Szabadics et al., 2006). To elicit monosynaptic GABA release from perisomatic inhibitory afferents, we electrically stimulated CA3 stratum pyramidale layer in close proximity (∼100 µm) of the recorded CA3 pyramidal cells. In ACSF + NBQX + AP5 conditions, we found that stimulation of CA3 stratum pyramidale was sufficient in eliciting APs in CA3 pyramidal neurons (Fig. 1*H*). In seven out of 12 cells, in which APs were triggered by electrical stimulation, the average AP threshold was lower (−56.2 ± 3.6 mV, *n* = 7 cells, *t*_(6)_ = 4.83, *p* = 0.003, paired *t* test) than the AP threshold obtained by somatic current injection (−33.0 ± 1,2 mV, *n* = 12 slices from three animals). However, it was still significantly higher as compared to those observed during light-induced (*p* = 0.02, *t*_(11)_ = 2.71, Student’s *t* test) or pipette (*p* = 0.04, *t*_(13)_ = 2.22, Student’s *t* test) chloride loading. Based on these experiments, we concluded that synaptically released GABA can reduce the AP threshold, perhaps due to moderate increase of intracellular chloride concentration derived simultaneously from the recording pipette and via GABA_A_ receptors. When intracellular chloride concentration rises even further as a result of chloride loading from high-chloride-containing recording pipette or eNpHR3.0 activation, the APs seem to be elicited at even lower threshold.

### Network activity is intensified during light illumination

**Figure 2.**
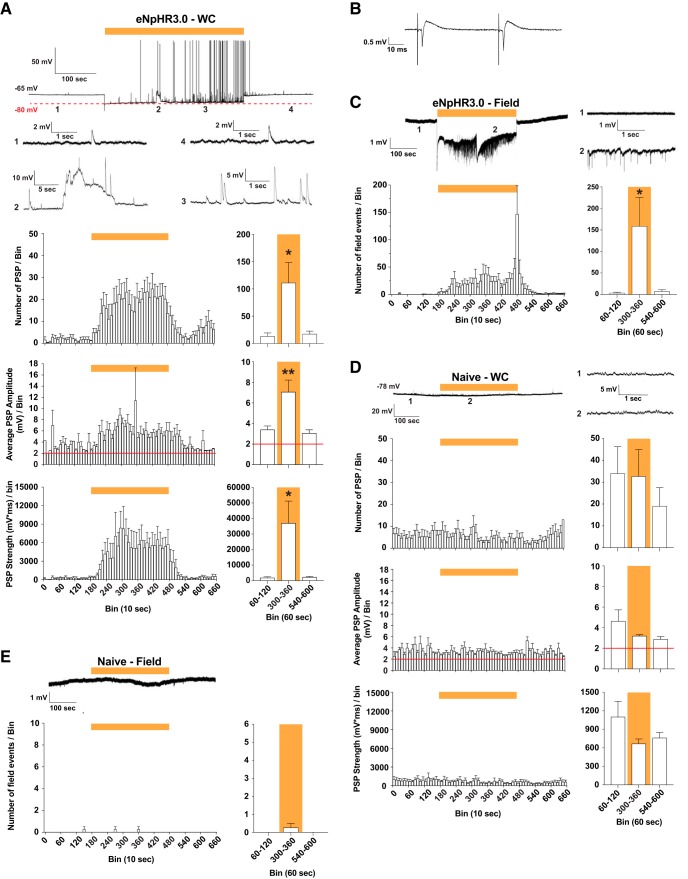
Long-term hyperpolarization using eNpHR3.0 renders the CA3 network into a hyperexcitable condition. ***A***, Whole-cell patch-clamp (WC) recordings of individual CA3 pyramidal cells were performed in physiologic ACSF. Top trace shows the entire recording of a representative cell at its RMP in current-clamp mode (0 pA). Light was applied for 5 min. Various periods (1-4) from the same trace are magnified and shown below. The top part of the APs is truncated in magnified trace 2. PSPs are shown in bins (10 and 60 s) and quantified by number (*F*_(2,13)_ = 5.17, *p* = 0.02), amplitude (*F*_(2,13)_ = 7.64, *p* = 0.006), and strength (*F*_(2,13)_ = 4.81, *p* = 0.03), *n* = 6 slices from three animals. ***B***, For field recordings, the placement of the electrode in stratum pyramidale CA3 was guided by electrical stimulation before the light experiment. ***C***, Same as ***A***, but for field recordings in eNpHR3.0-transfected slices, *F*_(2,24)_ = 5.20, *p* = 0.01, *n* = 9 slices from five animals. ***D***, Same as ***A***, but performed in slices from naïve animals (i.e., no eNpHR3.0 expression), number: *F*_(2,12)_ = 0.55, *p* = 0.59; amplitude: *F*_(2,12)_ = 1.97, *p* = 0.18; strength: *F*_(2,12)_ = 2.00, *p* = 0.18, *n* = 5 slices from two animals. ***E***, Same as ***C***, but performed in slices from naïve animals, *F*_(2,9)_ = 1.0, *p* = 0.41, *n* = 4 slices from two animals. All data are shown as mean ± SEM for 10- and 60-s bins. All comparisons for 60-s bins were made by one-way ANOVA with Dunnett’s *post hoc* test with prelight period serving as the control, *p < 0.05, **p < 0.01. Yellow bar indicates the period when light was applied, and red line in ***A***, ***D*** shows the detection threshold.

During the light-induced chloride-loading experiments in normal ACSF, we also observed multiple high-frequency depolarizing PSPs arising in CA3 neurons. During the course of light illumination (at 0 pA holding), multiple depolarizing PSPs appeared within 10-30 s. These PSPs increased both in frequency and amplitude over time, resulting in strong depolarization of the cells, which continued throughout the 5-min light application (Fig. 2*A*). The PSPs (Fig. 2*A*, trace 3) and slow depolarizations (Fig. 2*A*, trace 2) stopped almost immediately once the light was turned off. These PSPs most likely reflected activation of surrounding neuronal network synaptically connected to the recorded cell, and contributing to the hyperexcitability of individual neurons. Without light illumination, no aberrant changes in RMP were detected, and frequencies and amplitudes of PSPs remained stable and unaltered over time (Fig. 2*A*, trace 1).

Next, we confirmed that the light-induced activation of afferent synaptic inputs in the recorded cells was not due to any damage incurred by recording pipette. To this end, we performed a set of recordings as above while simultaneously recorded field potentials within the same CA3 subfield. The field-recording electrode was positioned into the CA3 pyramidal cell layer to a depth where electrically evoked fEPSPs could easily be elicited (Fig. 2*B*). In the submerged recording chamber, spontaneous fEPSP in adult hippocampal slices perfused with physiologic ACSF occur very sporadically, unless artificially provoked, for example by omitting Mg^2+^, applying drugs (e.g., 4-aminopyridine) or by electrical stimulation. In accordance, we detected only few fEPSPs (Fig. 2*C*). However, while applying light for 5 min, fEPSPs appeared within 10-30 s and were maintained at high frequency during the illumination period. When light was turned off, a wave of fEPSPs was observed, most likely reflecting synchronized rebound discharges in a network of neurons (Raimondo et al., 2012; Chuong et al., 2014). In the following period, occurrence of fEPSPs gradually declined back to baseline levels (Fig. 2*C*). The results thereby indicated that both PSPs and APs observed in individual neurons during light were not due to the whole-cell recording configuration, but most likely occurred in all surrounding eNpHR3.0-expressing cells not connected to the pipette.

To exclude the possibility that the aberrant network activity was provoked by increased local temperature in the slices caused by heat transfer by light illumination, we performed identical experiments in naïve slices (without eNpHR3.0 expression). Under these conditions, both RMP and appearance of PSPs (i.e., frequency and amplitude) in individual CA3 pyramidal cells remained stable and unaffected by light (Fig. 2*D*). Also, occurrence of spontaneous APs was not detected. A similar outcome was observed for field recordings, where we only observed few fEPSPs, and their occurrence was not influenced by light (Fig. 2*E*). We also excluded that downregulation of KCC2 chloride transporter (extruding chloride from the cells) was not directly involved in the observed phenomenon. In the presence of NBQX + AP5 when adding the selective KCC2 antagonist, VU0463271 (Sivakumaran et al., 2015), to slices from naïve animals (*n* = 14 slices from three animals), the RMP became slightly, but significantly, depolarized (4.63 ± 0.68 mV, *t*_(13)_ = 7.10, *p* = 0.001, Student’s paired *t* test). However, PSPs and slow depolarizations, as seen during the light application (Fig. 2*A*, trace 2-3), were not detected.

### Aberrant network activity during chloride loading is mediated by GABA_A_ receptors

**Figure 3.**
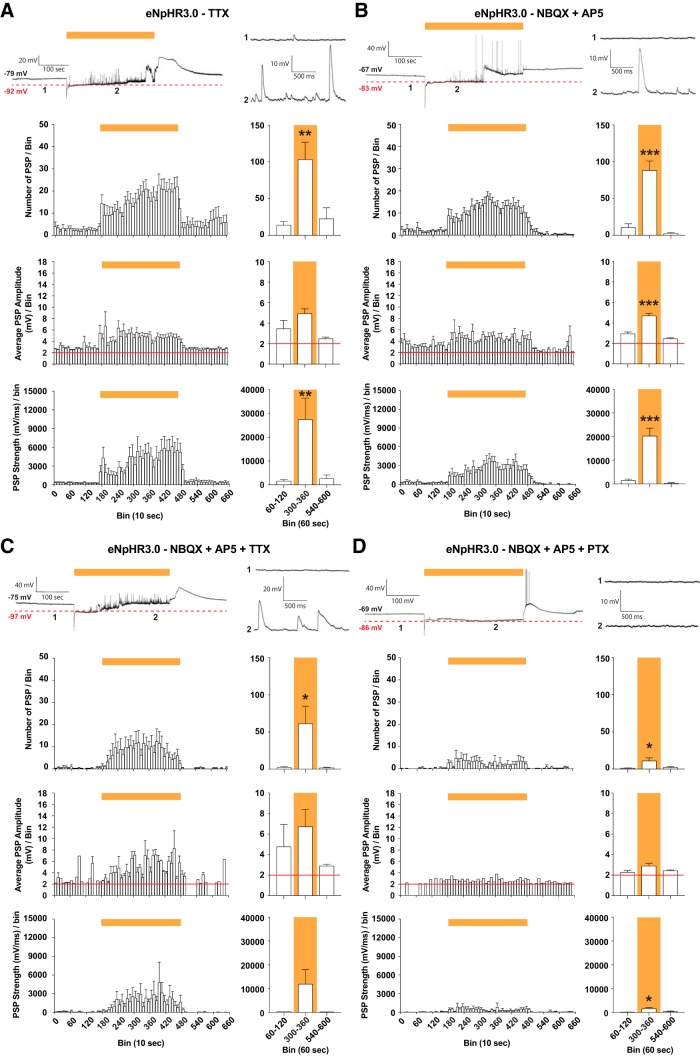
GABAergic neurotransmission is a main trigger of the hyperexcitable condition caused by eNpHR3.0 activation. ***A***, Whole-cell patch-clamp recording of CA3 pyramidal cells were performed in physiologic ACSF together with TTX, and light was applied for 5 min. Top, Representative trace with magnified traces (1-2) shown on the right. PSPs are shown in bins (10 and 60 s) and quantified by number (*F*_(2,24)_ = 8.87, *p* = 0.0013), amplitude (*F*_(2,20)_ = 3.29, *p* = 0.058), and strength (*F*_(2,24)_ = 7.57, *p* = 0.0028); *n* = 9 slices from four animals. ***B***, Same as ***A***, but performed in physiologic ACSF together with NBQX + AP5, number: *F*_(2,21)_ = 34.12, *p* < 0.0001; amplitude: *F*_(2,17)_ = 33.1, *p* < 0.0001; strength: *F*_(2,21)_ = 32.47, *p* < 0.0001, *n* = 8 slices from three animals. ***C***, Same as ***A***, but performed in physiologic ACSF together with NBQX + AP5 + TTX, number: *F*_(2,10)_ = 4.77, *p* = 0.035; amplitude: *F*_(2,6)_ = 1.01, *p* = 0.42; strength: *F*_(2,10)_ = 2.68, *p* = 0.12, *n* = 5 slices from two animals. ***D***, Same as ***A***, but performed in physiologic ACSF together with NBQX + AP5 + PTX, number: *F*_(2,18)_ = 4.52, *p* = 0.027; amplitude: *F*_(2,9)_ = 1.67, *p* = 0.24; strength: *F*_(2,18)_ = 5.82, *p* = 0.011, *n* = 7 slices from three animals. All data are shown as mean ± SEM in 10- and 60-s bins, as indicted, and analyzed using one-way ANOVA with Dunnett’s *post hoc* test with prelight period serving as the control, *p < 0.05, **p < 0.01, ***p < 0.001. Yellow bar indicates the period when light was applied, whereas red line shows the detection threshold.

Based on our observations that light-induced increased frequency of PSPs in individual neurons was a result of elevated network activity, we hypothesized that these aberrant events in eNpHR3.0-expressing CA3 neurons was due to activation of GABA_A_ receptors. To test this hypothesis, we first exposed hippocampal slices to 5-min light illumination during whole-cell recordings from CA3 neurons while adding TTX into the perfusion solution. TTX is a potent voltage-gated sodium channel antagonist that effectively blocks the generation of APs. Under these conditions, we detected an overall decrease of PSP activity (i.e., frequency and amplitude), but light still evoked aberrant depolarizing activity (Fig. 3*A*). A similar outcome was obtained when blocking fast glutamatergic transmission by adding NBQX and AP5 to ACSF (Fig. 3*B*) or applied these drugs together with TTX (Fig. 3*C*). Only after adding the GABA_A_ receptors antagonist, PTX, to ACSF the activity pattern was almost completely normalized to pre- and post-light periods (Fig. 3*D*). Collectively, these pharmacological experiments suggest that the depolarizing PSPs and slow depolarizing waves during eNpHR3.0 activation are predominantly generated by chloride flow through GABA_A_ receptors. It is not clear in the TTX condition, however, how increased intracellular chloride activates GABA_A_ receptors. Since eNpHR3.0 expression was driven by the pan-neuronal human synapsin 1 promoter, the transgene was most likely also expressed in synaptic terminals of GABAergic neurons, implying that light may depolarize those and induce GABA release even when APs are blocked by TTX.

## Discussion

Here, we demonstrate that chloride loading by long-lasting optogenetic activation of eNpHR3.0 in the hippocampal CA3 neurons leads to significantly lowered AP threshold and aberrant excitatory activity in individual neurons and networks. Lowered AP threshold was not a result of the optogenetic manipulation in itself, as similar results were obtained by loading neurons with chloride via a high-chloride-containing solution in the patch pipette. Likewise, the aberrant activity observed in individual neurons was not due to the patch pipette solution, since field recordings revealed similar activity in the whole population of neurons expressing eNpHR3.0, not affected by the patch pipette.

How can chloride load decrease the AP threshold? Hyperpolarization by eNpHR3.0 activation per se cannot explain this phenomenon, since measuring the AP threshold from a prior hyperpolarized membrane potential had no effect. Previously, voltage-independent sodium channels have been implicated in changing the AP threshold in interneurons (Lu et al., 2014). Also, calcium-dependent chloride channels (CaCCs), which are expressed in CA3 pyramidal neurons, have been shown to increase coupling between EPSP and AP generation with chloride loading (Huang et al., 2012). Further investigations are required to delineate the exact mechanisms by which chloride overload alters AP threshold.

The present finding that the AP threshold can substantially change in situations with chloride overload is of particular interest, since, to our knowledge, this has not been described previously and may represent a novel mechanism of regulating cellular and network excitability. The mechanism could be a contributing factor to network excitability during developmental stages as well as pathologic conditions of adult brain, e.g., epilepsy and other hyperexcitability states (Kullmann and Waxman, 2010), when intracellular concentration of chloride is markedly higher compared to normal physiologic conditions.

What are the mechanisms behind the slow aberrant depolarizing events seen in CA3 neurons (Fig. 2*A*, trace 2) during light illumination? Previous studies have shown that light-activation of eNpHR3.0 for a few seconds can depolarize neurons via GABA_A_ receptors due to intracellular chloride accumulation (Raimondo et al., 2012; Alfonsa et al., 2015; Mahn et al., 2016). Such accumulation causes a substantial positive shift in the GABA-induced chloride-reversal potential proportional to the charge transfer, in the magnitude of 20-25 mV for short light pulses (<20 s), and converts hyperpolarizing responses of synaptically released GABA to being depolarizing (Raimondo et al., 2012; Alfonsa et al., 2015). In our case, the GABA-induced depolarizing effects were much more pronounced, which could be explained by substantially longer (5 min) activation of eNpHR3.0 with subsequent increased chloride accumulation decreasing the reversal potential further. Since inadequate chloride extrusion through KCC2 appeared not to be critical for the slow depolarizations, it could tentatively involve voltage-gated volume-sensitive outwardly rectifying (VSOR) chloride channels (Inoue and Okada, 2007), CaCCs (Huang et al., 2012), and/or chloride-channels (ClCs; Jentsch et al., 2002; Rinke et al., 2010). Among these, the ClC-2 subtype, is perhaps the most relevant in our experimental conditions. It is abundantly expressed in the CA3 region (Cortez et al., 2010) and activated at membrane potentials more negative than the chloride-reversal potential (Staley et al., 1996). Consequently, when eNpHR3.0 is activated, the significant rise in intracellular chloride accompanied with a hyperpolarized membrane potential, should open ClC-2, and hence counteract chloride accumulation and hyperpolarization. Because ClC-2, including eNpHR3.0, expression might be unequally distributed along various compartments (i.e., somata, axon initial segment, dendrites), we cannot exclude that the membrane potential measured at the cell soma via patch pipette, actually reflect the true membrane potential elsewhere in the cell. Nonetheless, since slow depolarizations were absent when PTX was added to ACSF, it could also imply that they were driven by accumulated extracellular GABA released from interneurons expressing eNpHR3.0.

In summary, we here demonstrate that chloride accumulation in hippocampal CA3 pyramidal neurons not only transforms GABAergic synaptic transmission into aberrant depolarizing excitation, it also decreases the AP threshold. The latter novel finding could further exacerbate network excitability in conditions where reverse chloride homeostasis due to altered expression of chloride transporters occurs, for example at early stages of brain development, and pathophysiological conditions such as epilepsy (Chen et al., 2017; Moore et al., 2017). These findings may also have implications in designing and developing therapeutic approaches aimed at counteracting hyperexcitable states by optogenetic strategies (Choy et al., 2017).

## Synthesis

Reviewing Editor: Alfonso Araque, University of Minnesota Decisions are customarily a result of the Reviewing Editor and the peer reviewers coming together and discussing their recommendations until a consensus is reached. When revisions are invited, a fact-based synthesis statement explaining their decision and outlining what is needed to prepare a revision will be listed below. The following reviewer(s) agreed to reveal their identity: Aristea Galanopoulou, Kevin Staley

Reviewers indicate that authors have adequately addressed previous concenrrns in the current version of the manuscript.

There are no further scientific comments, but authors should check the manuscript to be proofed for typos, e.g.

- title: change increase to increases

- page 10, line 208: “sufficient of elicit": change to sufficient in eliciting..."
